# Supplementary material for: Endothelial-erythrocyte glycocalyx exchange enables liquid biopsies of endothelial function
Source: Nat Commun. 2026 May 12;17:3568. doi: 10.1038/s41467-026-71848-4 (PMC13168509; doi:10.1038/s41467-026-71848-4)
Supplement: Supplementary file 1 — Supplementary Information [file 41467_2026_71848_MOESM1_ESM.pdf]

Endothelial-erythrocyte glycocalyx exchange enables liquid biopsies of endothelial function

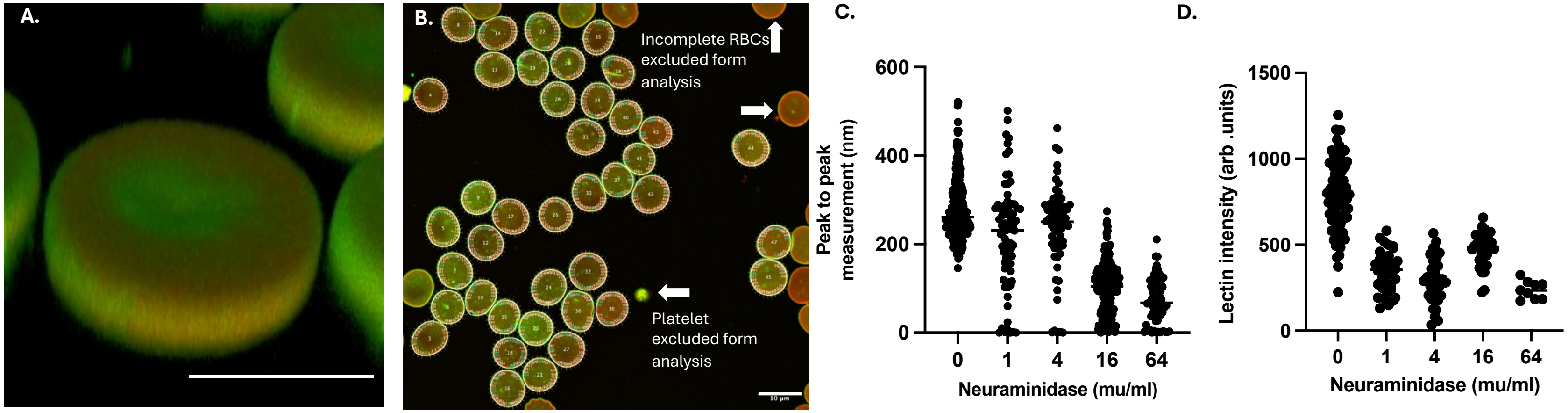

Supplementary Fig 1. Automated software identifies RBC and measures RBCGlx depth and intensity A. A 3D reconstruction generated from a z-stack of confocal images of RBCs labelled with LEL lectin (green) and R18 (red). Note the RBC morphology is well preserved during the fixation and staining processes and that LEL lectin covers the RBC surface on heathy donor RBC. (Bar = 5µm) **B**. A blood smear image where the AI software package has identified and numbered all RBC and placed line profiles tangential to the cell membrane ready for analysis whilst ignoring RBC partially imaged at the field periphery and platelets. (Bar = 10µm) **C**. Automated analysis of RBC labelled with LEL from 5 experiments highlighting the RBC population 'peak to peak' distribution at baseline and following exposure to low levels of neuraminidase (a sialic acid depleting enzyme). Each point on the graph represents the median 'peak to peak' value for RBC. Data highlight that the effect of 1mU/ml neuraminidase on the RBC population can be detected after 30 minutes exposure (one way ANOVA $p<0.0001$). **D** The automated software also provides measures of peak lectin signal intensity at the RBC membrane. Again, we detected significant changes following 1mU/ml neuraminidase exposure for 30 minutes. Analysis by one way ANOVA ($p<0.0001$)

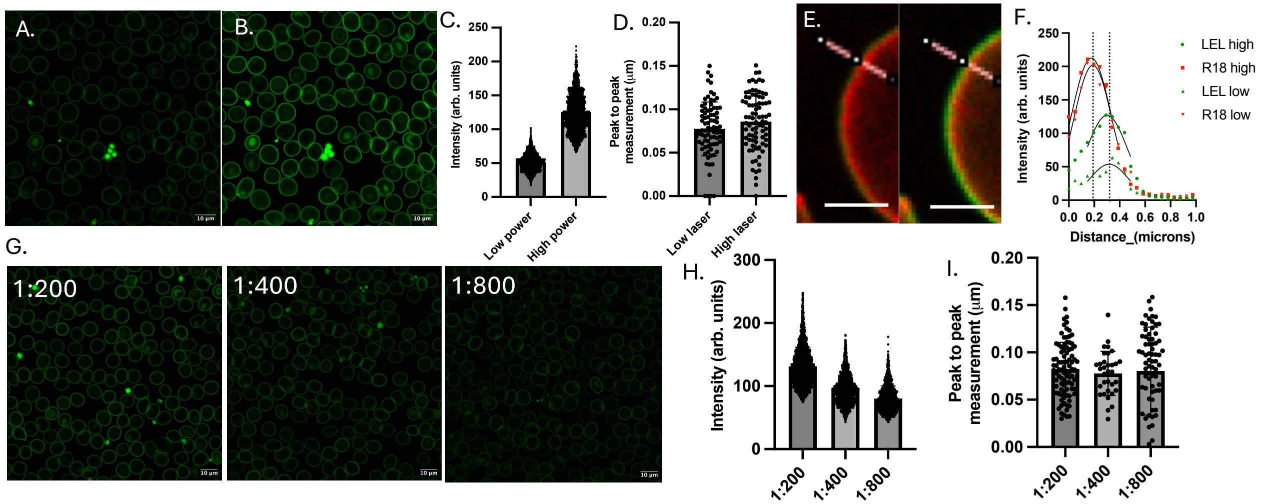

Supplementary Fig 2. 'Peak to peak' imaging provides a robust measure of RBCGlx. A. An RBC smear labelled with LEL lectin and R18 and imaged with low laser power (for representative images only the LEL signal is shown for ease of visualisation) **B.** The identical area imaged using an optimized laser power. **C.** Laser power directly affects the RBC LEL signal intensity measured on the RBC surface. (n=84 RBC, t-test p<0.0001) **D. '**Peak to peak' assessment of the same images confirms that the laser power has no effect on the peak LEL or R18 signal locations. (n=84 RBC, t-test, p=0.093) **E.** Close up images of a single RBC (red = cell membrane, green = glycocalyx) where low and optimal laser power have been used. (Bar = 1μm) **F**. Illustrated line profiles generated by the regions of interest in (E.) Note that the center of the lectin associated Gaussian peaks align despite the variable laser intensity used. **G.** RBC smear stained with LEL diluted 1:200 (standard concentration), 1:400 and 1:800 and imaged using fixed settings. **H.** RBC cell surface LEL intensity (by line profile) varies with variable lectin dilution. (n=50 RBC, one way ANOVA p=<0.0001) **I.** 'Peak to peak' assessment of the same images confirmed that variable lectin concentrations do not affect the 'peak to peak' measure (one way ANOVA p=0.79).

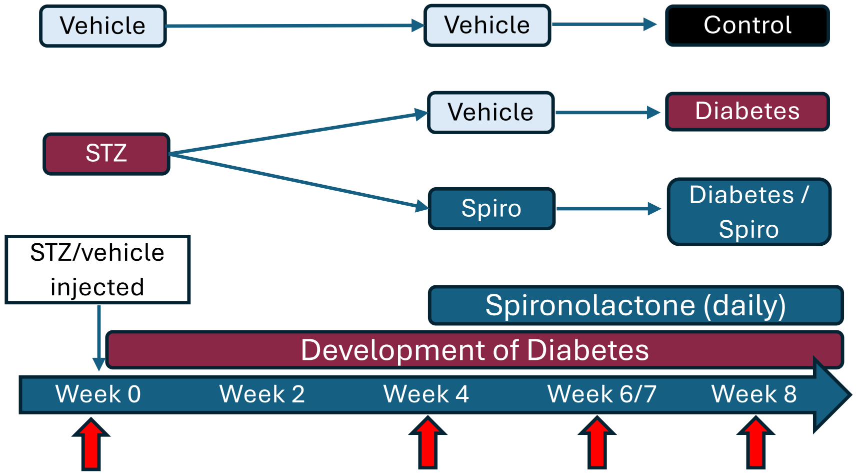

Supplementary Fig 3. Diabetic rat study protocol. Male Wistar rats (150–200 g, Charles River Laboratories) were maintained in a conventional facility (21°C–24°C and 12:12 hour light/dark cycle). Randomized rats were injected intraperitoneally with 50 mg/kg STZ (S0130; Sigma-Aldrich) (25 mg/mL in 10 mM sodium citrate, pH 4.5). Four weeks after STZ injection, spironolactone (spiro) was given for 28 days (S3378; Sigma-Aldrich) at 50 mg/kg made up in corn oil (C8267; Sigma-Aldrich). Blood glucose level was measured 3 weeks after STZ by tail-tip blood droplet analysis using a glucometer (Accu-Chek Aviva; Roche), and rats with glycemia ≥ 15 mmol/L were considered diabetic and included in the study. Rats were culled 8 weeks after STZ/vehicle injection. Comparisons were made with vehicle-treated rats (10 mM sodium citrate [pH 4.5]). RBCGlx was measured on blood samples taken from the tail vein at 4 time points indicated by the red arrows.

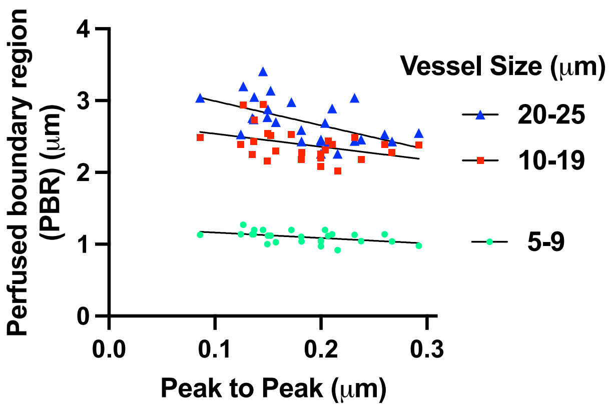

Vessel size	5-9	10-19	20-25	Total PBR 5-25
R squared	0.2053	0.156	0.3031	0.2512
P value	0.0201	0.0458	0.0029	0.0077

Supplementary Fig 4. RBCGlx 'peak to peak' measure corelated with GlycoCheck™ PBR in all vessel size ranges. The median RBCGlx depth for individual pregnant women in the first trimester corelated (linear regression) with the sublingual PBR measurement in all measured vessel sizes. Relevant R squared values and P values are also shown. (n=individual participant in the study) (blue tringles = PBR in vessels 20-25µm, red squares = PBR in vessels 10-19µm, green circles = PBR in vessels 5-9µm)

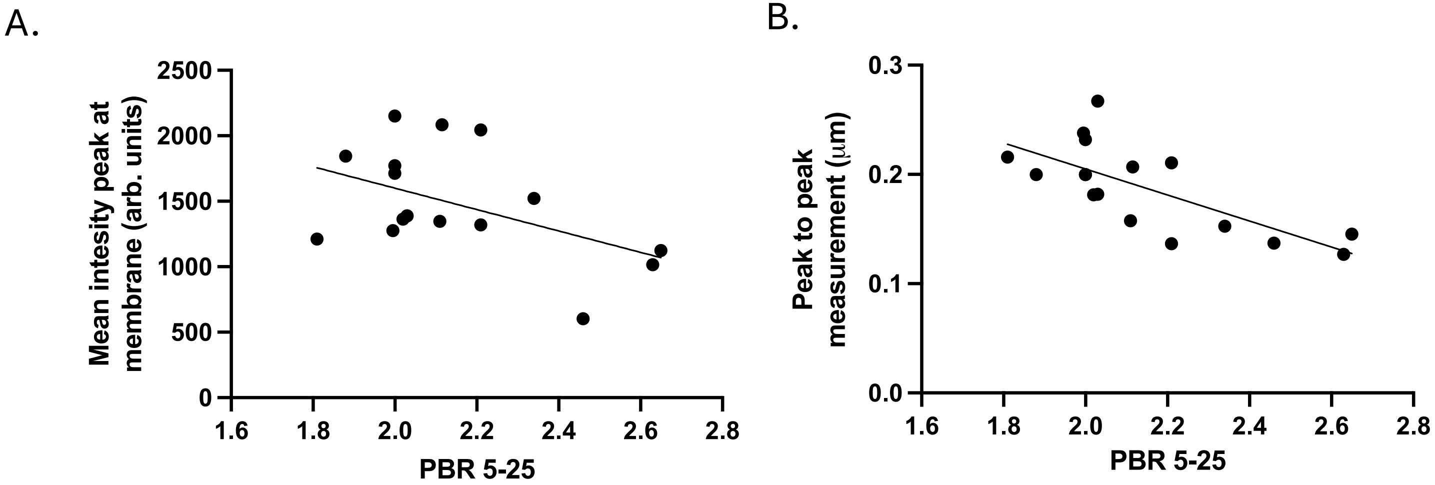

Supplementary Figure 5. Comparison of 'peak to peak' and intensity-based measurements of the RBC glycocalyx with sublingual GlycoCheck™ based assessment of eGlx integrity in healthy pregnant women. A. Blinded images of RBC smears, labelled with LEL, and taken using fixed settings on a single microscope were analysed using line profiles tangential to the cell membrane to record the peak value minus the level of localized background signal around each RBC circumference. The measurements for each RBC analysed were subsequently used to generate a mean intensity value (arbitrary unit (arb .units) for each patient (displayed n=17) r=-0.48,R squared 0.2339, p=0.0492. **B.** For comparison the matched 'peak to peak' values are shown for the same trial participants (n=17) r-0.72, R squared 0.52, p=0.001. Both analysis methods produced a significant correlation with the PBR value. Power calculations suggest 32 patients are needed for future studies studying the correlation between lectin intensity and PBR or 13 patients for 'peak to peak' based comparisons.

Characteristic	DISCOVER	Control
Age	60.5 (34-93)	59 (32-76)
Sex (female)	11/23	3/6
PCR proven COVID-19 at admission	23/23	0/6
Diabetes	7/23	2/6
Heart Disease	3/23	0/6
Chronic lung disease	7/23	1/6
Severe liver disease	3/23	0/6
Severe kidney impairment (eGFR<30 or dialysis)	2/23	0/6
Hypertension	2/23	2/6
HIV positive	1/23	0/6

Supplementary Fig 6. Demographic data for patients where RBCGlx depth was measured as part of the DISCOVER study. Patients and controls were simultaneously recruited from the 18th September 2020 until the 11th of November 2020

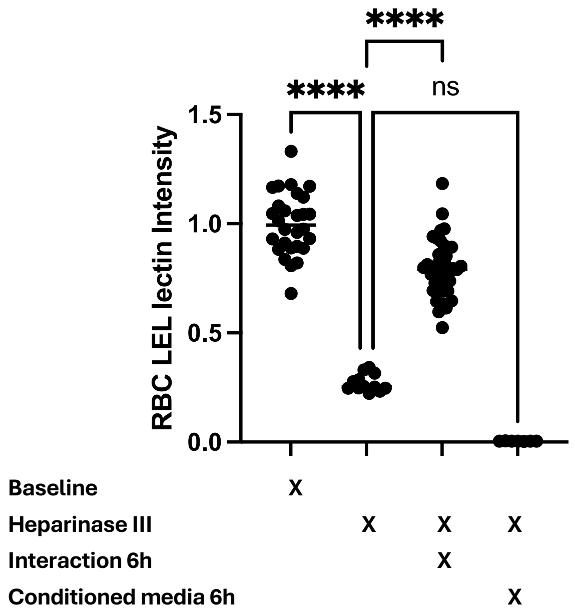

Supplementary Fig 7. RBC LEL surface signal intensity normalized to untreated RBC. Heparinase III treated RBC, subsequently labelled with LEL lectin, have reduced surface signal intensities compared to cells labelled at baseline. Again, endothelial interaction (5dyn) significantly increased LEL lectin intensity after 6 hours. In contrast 6 hours exposure to endothelial cell conditioned media had no effect on LEL RBC surface intensity (displayed data = RBC from 3 experimental repeats, analysis by one way ANOVA with Tukey's comparisons).

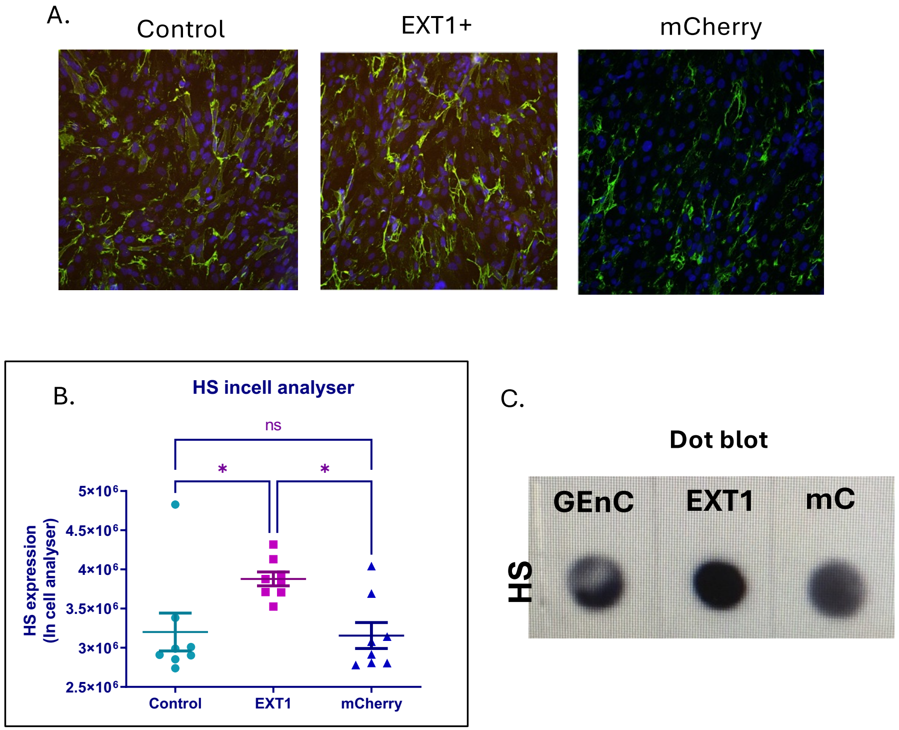

Supplementary Fig 8. – **EXT1+ cells over express HS A**. Transduction of endothelial cells with lentivirus expressing EXT1-Myc increased surface HS expression on endothelial monolayers visibly (Blue = nuclei (DAPI), Green = HS). HS was labelled using anti HS antibody (BIO-RAD, cat no1698). **B/C** Automated image acquisition and analysis of labelled endothelial cultures and dot blotting confirmed EXT1 overexpression on endothelial monolayers increased HS expression. Analysis by one way ANOVA with Tukey's comparison n = 8 (culture replicate) data = mean +/- SEM (turquoise circles = control cultures, pink squares = EXT1 over expressing cultures, blue triangles = mCherry control)

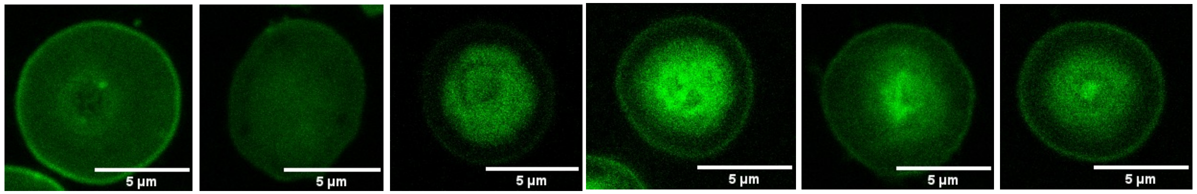

Supplementary Fig 9. Changes in endothelial cell heparan sulphate expression can be detected on RBC. Following neuraminidase (300mU/ml 1hour) LEL lectin 'peak to peak' thickness was significantly reduced on human RBC. Representative images of RBC labelled with LEL lectin highlight that after 1 hour interaction with EXT1+ endothelial cells RBC recovery is visible but not after interaction with wild type cells. After 14 hours interaction no differences were visible (or detected) between RBC interacted with EXT1+ cells and wild type endothelial cells.

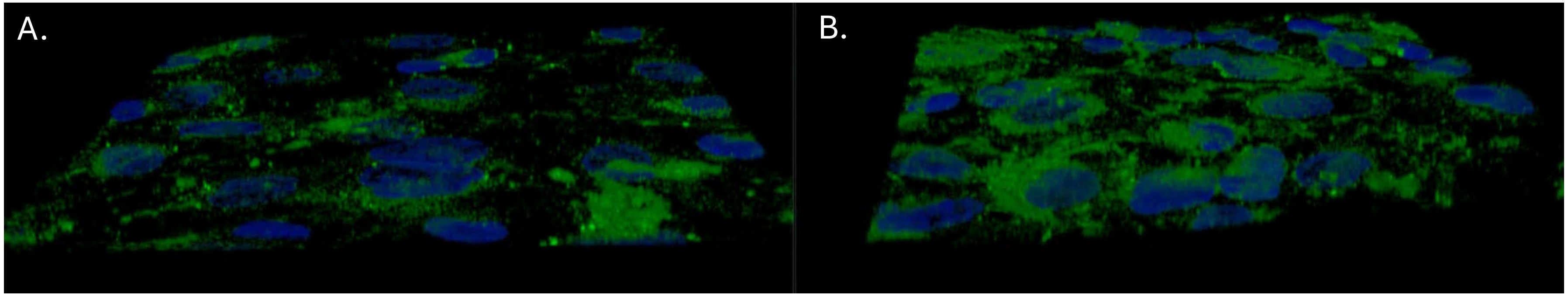

Supplementary Fig 10. **Lectin transfer from RBC is increased following interaction with damaged endothelial monolayers**. 3D z-stack reconstructions of endothelial monolayers labelled with DAPI (blue) and WGA lectin (green). Both monolayer were interacted with washed WGA labelled RBC for 6hours. **A**. Control endothelial cells (grown under standard conditions) demonstrate WGA-FITC signal on their surface (green). **B.** Enzymatically damaged endothelial cells, again interacted with washed WGA labelled RBC for 6hours, demonstrate enhanced surface signal (green) consistent with increased transfer of lectin or lectin bound glycocalyx components from the RBCs.

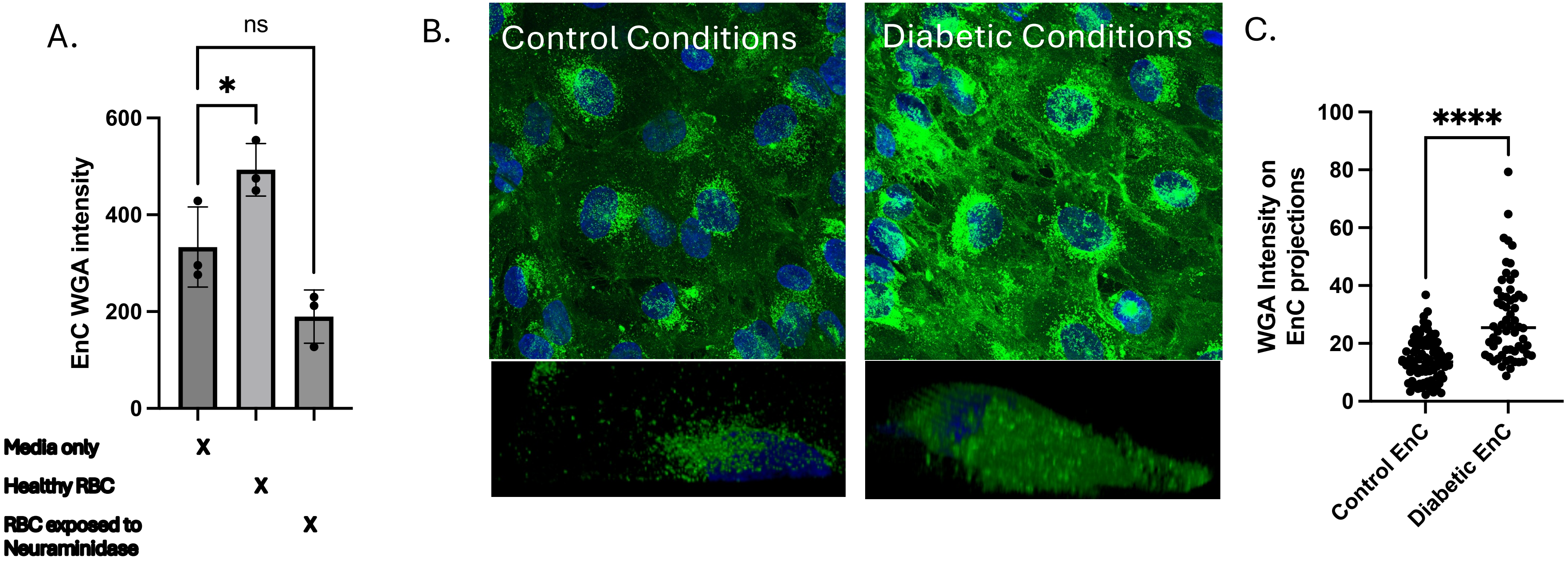

Supplementary Fig 11. **Red blood cell - endothelial cell interaction can result in endothelial glycocalyx repair in diabetic models, an effect that is dependent on the RBC Glx. A.** Endothelial monolayers cultured under diabetic conditions were exposed to 5dyn shear for 6h. Where healthy RBC were added to the cell media a significant increase in the endothelial cell surface WGA lectin signal intensity was seen at the experimental end compared to cells exposed only to flowing media. No increase in the WGA intensity was seen when diabetic endothelial monolayers were exposed to washed RBC with a depleted glycocalyx (Neuraminidase 300mU/ml 1hr) confirming the RBC glycocalyx was essential to this repair mechanism (n=3 biological replicates per group, analysis by ANOVA with Tukey's comparison, mean +/- SD) **B.** A separate series endothelial monolayers were interacted with washed WGA labelled RBC for 6hours. 3D z-stack reconstructions (maximum projected values) of endothelial monolayers labelled directly with DAPI only (blue). WGA lectin (green) transfers from the RBC surface onto the surface of endothelial cells. The insets confirm surface expression over the nucleus. Increased lectin transfer is seen from the labelled RBC onto endothelial cells cultured under diabetic conditions compared to control conditions. **C.** Quantification of the intensity of the transferred lectin (measured on the endothelial cells' surface) suggested that RBC and endothelial glycocalyx exchange continues to occur in under diabetic conditions with the net direction of transfer favoring repair of either structure if individually damaged (n=5, analysis by two-tailed T-Test, $p<0.001$).

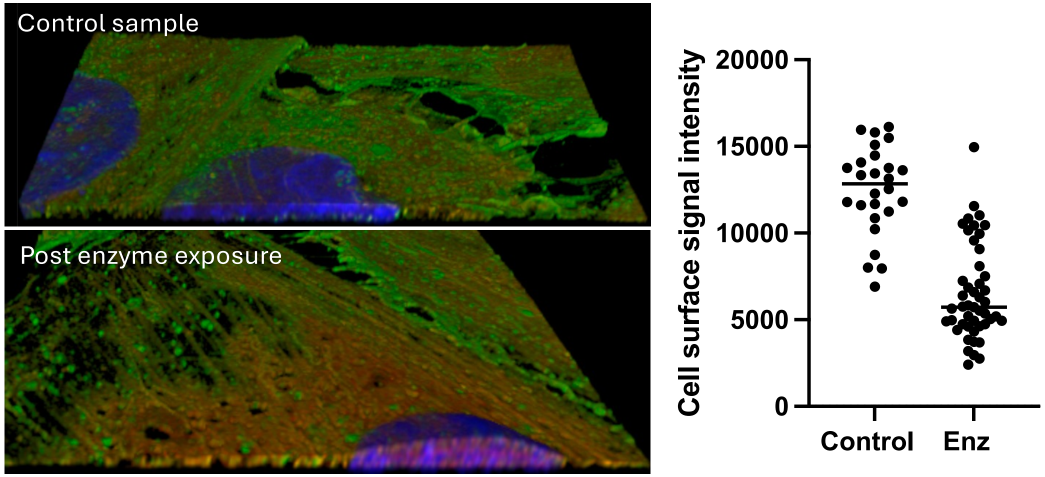

Supplementary Fig 12 Confirmation ManNAz integrates into eGlx and can be depleted by exogenous enzymes within 2 hours. Endothelial cells fed ManNAz supplemented media integrate the 'Click' label into sialic acid (SA). SA is a major component of the glycocalyx. Enzyme exposure (heparinase III 1u/ml (Sigma H8891), neuraminidase 30mU/ml (Roche, 11 585 886 001) significantly reduce cell surface 'Click' label, suggesting loss of cell surface SA relative to control cells.
