# Supplementary material for: Endothelial-erythrocyte glycocalyx exchange enables liquid biopsies of endothelial function
Source: Nat Commun. 2026 May 12;17:3568. doi: 10.1038/s41467-026-71848-4 (PMC13168509; doi:10.1038/s41467-026-71848-4)
Supplement: Supplementary file 2 — Reporting summary [file 41467_2026_71848_MOESM2_ESM.pdf]

nature portfolio

Corresponding author(s): Dr Matthew Butler
Last updated by author(s): Nov 7, 2025

Reporting Summary

Nature Portfolio wishes to improve the reproducibility of the work that we publish. This form provides structure for consistency and transparency in reporting. For further information on Nature Portfolio policies, see our Editorial Policies and the Editorial Policy Checklist.

Statistics

For all statistical analyses, confirm that the following items are present in the figure legend, table legend, main text, or Methods section.

n/a	Confirmed	
☐	☒	The exact sample size (*n*) for each experimental group/condition, given as a discrete number and unit of measurement
☐	☒	A statement on whether measurements were taken from distinct samples or whether the same sample was measured repeatedly
☐	☒	The statistical test(s) used AND whether they are one- or two-sided<br>*Only common tests should be described solely by name; describe more complex techniques in the Methods section.*
☐	☒	A description of all covariates tested
☐	☒	A description of any assumptions or corrections, such as tests of normality and adjustment for multiple comparisons
☐	☒	A full description of the statistical parameters including central tendency (e.g. means) or other basic estimates (e.g. regression coefficient) AND variation (e.g. standard deviation) or associated estimates of uncertainty (e.g. confidence intervals)
☐	☒	For null hypothesis testing, the test statistic (e.g. *F*, *t*, *r*) with confidence intervals, effect sizes, degrees of freedom and *P* value noted<br>*Give P values as exact values whenever suitable.*
☒	☐	For Bayesian analysis, information on the choice of priors and Markov chain Monte Carlo settings
☒	☐	For hierarchical and complex designs, identification of the appropriate level for tests and full reporting of outcomes
☒	☐	Estimates of effect sizes (e.g. Cohen's *d*, Pearson's *r*), indicating how they were calculated

Our web collection on statistics for biologists contains articles on many of the points above.

Software and code

Policy information about availability of computer code

Data collection	No software was used.
Data analysis	Where automated analysis was employed RBC within images were detected using a custom StarDist model. Image J (FIJI) Intensity profiles perpendicular to object surfaces were measured using a custom workflow created using the ModularImageAnalysis (MIA) plugin

For manuscripts utilizing custom algorithms or software that are central to the research but not yet described in published literature, software must be made available to editors and reviewers. We strongly encourage code deposition in a community repository (e.g. GitHub). See the Nature Portfolio guidelines for submitting code & software for further information.

Data

Policy information about availability of data

All manuscripts must include a data availability statement. This statement should provide the following information, where applicable:

- Accession codes, unique identifiers, or web links for publicly available datasets
- A description of any restrictions on data availability
- For clinical datasets or third party data, please ensure that the statement adheres to our policy

All summary data is included within the manuscript. There are no large datasets and all raw data can be provided on request.

Research involving human participants, their data, or biological material

Policy information about studies with human participants or human data. See also policy information about sex, gender (identity/presentation), and sexual orientation and race, ethnicity and racism.

Reporting on sex and gender	The DISCOVER study enrolled male and female participants and the numbers are provided in supplemental figure 5<br>All participants in the SPADE study were female (pregnancy was an inclusion criteria for this study)
Reporting on race, ethnicity, or other socially relevant groupings	N/A
Population characteristics	Demographic details are suppled in supplemental figure 5
Recruitment	Both studies recruited from Bristol hospitals. DISCOVER recruited patients with COVID 19 confirmed by a positive PCR result for SARS COV2. Blood samples were collected by research nurse from all consenting patients within the study period. No potential bias has been identified.<br>https://research-information.bris.ac.uk/ws/portalfiles/portal/289127273/DISCOVER_BMJem_resub2_CLEAN.pdf<br>The SPADE study was a prospective longitudinal cohort study which enrolled pregnant women with T1DM and T2DM and normoglycaemic (NG) controls. All women recruited within the study period where the blood sample could be processed within 4 hours were included. Again no potential for bias was identified.
Ethics oversight	Ethics<br>DISCOVER approval via South Yorks REC: 20/YH/0121, CRN approval no: 45469<br>SPADE approval National Research Ethics Service (NRES) committee South-West – (ref 06/Q2006/54) (ref OG/2006/2352)

Note that full information on the approval of the study protocol must also be provided in the manuscript.

Field-specific reporting

Please select the one below that is the best fit for your research. If you are not sure, read the appropriate sections before making your selection.

☒ Life sciences ☐ Behavioural & social sciences ☐ Ecological, evolutionary & environmental sciences

For a reference copy of the document with all sections, see nature.com/documents/nr-reporting-summary-flat.pdf

Life sciences study design

All studies must disclose on these points even when the disclosure is negative.

Sample size	Sample size estimates were based on provisional experimental results
Data exclusions	No data were excluded from analysis.
Replication	Experimental replicates were included in all studies to confirm data reproducibility.
Randomization	Where animals were used each cage was randomized (to act as controls or have diabetes induced.) Diabetic animals were randomised again to receive either daily spironolactone or vehicle. A coin toss was use to allocate animal groups. During in vitro experiments culture plates were randomly used for each condition tested .
Blinding	Investigators were not blinded to allocations during the studies due to daily interventions eg spironolactone injection or ManNAz addition to cell media. However, all investigators were blinded at all stages of sample collection, processing, imaging and analysis.

Reporting for specific materials, systems and methods

We require information from authors about some types of materials, experimental systems and methods used in many studies. Here, indicate whether each material, system or method listed is relevant to your study. If you are not sure if a list item applies to your research, read the appropriate section before selecting a response.

Materials & experimental systems

n/a	Involved in the study	
☒	☐	Antibodies
☐	☒	Eukaryotic cell lines
☒	☐	Palaeontology and archaeology
☐	☒	Animals and other organisms
☒	☐	Clinical data
☒	☐	Dual use research of concern
☒	☐	Plants

Methods

n/a	Involved in the study	
☒	☐	ChIP-seq
☒	☐	Flow cytometry
☒	☐	MRI-based neuroimaging

Eukaryotic cell lines

Policy information about cell lines and Sex and Gender in Research

Cell line source(s)	The original source cells and methods are discussed in this publication https://www.kidney-international.org/article/S0085-2538(15)51704-3/fulltext.
Authentication	As above
Mycoplasma contamination	Cells are regularly screened for mycoplasma tested negative
Commonly misidentified lines (See ICLAC register)	*Name any commonly misidentified cell lines used in the study and provide a rationale for their use.*

Animals and other research organisms

Policy information about studies involving animals; ARRIVE guidelines recommended for reporting animal research, and Sex and Gender in Research

Laboratory animals	Species and strain and age data are included in the manuscript. Male Wistar rats (150–200 g, Charles River Laboratories) were maintained in a conventional facility (21°C–24°C and 12:12 hour light/dark cycle).<br><br>Endothelial specific EXT1 conditional knockout mice (EXT1(ECKO)) and litter mate control mice were generated on a C57BL/6 background by crossing Cdh5(PAC)-CreERT2 mice (Taconic) with EXT1-floxed (EXT1fl/fl) mice. In male Cdh5(PAC)-CreERT2;EXT1flox/flox mice (aged 6-10 weeks) gene excision was induced by tamoxifen IP injection (75mg/kg) for 5 consecutive days. Blood samples from Cdh5(PAC)-CreERT2;EXT1flox/flox mice and their littermate controls
Wild animals	The study did not involve wild animals
Reporting on sex	The breeding colony for the EXT1 KO mice was established to utilise male mice (induced with tamoxifen)<br>The samples taken from rats were taken from a historical study (https://insight.jci.org/articles/view/154164) where male rats were used.
Field-collected samples	No field samples were used in this study.
Ethics oversight	All animal protocols were approved by the UK Government Home Office and conformed to the Guide for the Care and Use of Laboratory Animals (National Academies Press, 2011)

Note that full information on the approval of the study protocol must also be provided in the manuscript.

Plants

Seed stocks	*Report on the source of all seed stocks or other plant material used. If applicable, state the seed stock centre and catalogue number. If plant specimens were collected from the field, describe the collection location, date and sampling procedures.*
Novel plant genotypes	*Describe the methods by which all novel plant genotypes were produced. This includes those generated by transgenic approaches, gene editing, chemical/radiation-based mutagenesis and hybridization. For transgenic lines, describe the transformation method, the number of independent lines analyzed and the generation upon which experiments were performed. For gene-edited lines, describe the editor used, the endogenous sequence targeted for editing, the targeting guide RNA sequence (if applicable) and how the editor was applied.*
Authentication	*Describe any authentication procedures for each seed stock used or novel genotype generated. Describe any experiments used to assess the effect of a mutation and, where applicable, how potential secondary effects (e.g. second site T-DNA insertions, mosiacism, off-target gene editing) were examined.*
